# Air entrapment and bubble formation during droplet impact onto a single cubic pillar

**DOI:** 10.1038/s41598-021-97376-3

**Published:** 2021-09-09

**Authors:** Weibo Ren, Patrick Foltyn, Anne Geppert, Bernhard Weigand

**Affiliations:** Institute of Aerospace Thermodynamics, University of Stuttgart, 70569 Stuttgart, Germany

**Keywords:** Engineering, Materials science, Mathematics and computing, Physics

## Abstract

We study the vertical impact of a droplet onto a cubic pillar of comparable size placed on a flat surface, by means of numerical simulations and experiments. Strikingly, during the impact a large volume of air is trapped around the pillar side faces. Impingement upon different positions of the pillar top surface strongly influences the size and the position of the entrapped air. By comparing the droplet morphological changes during the impact from both computations and experiments, we show that the direct numerical simulations, based on the Volume of Fluid method, provide additional and new insight into the droplet dynamics. We elucidate, with the computational results, the three-dimensional air entrapment process as well as the evolution of the entrapped air into bubbles.

## Introduction

Droplet impact onto surfaces is ubiquitous in nature and daily life. One can observe, for example, splashing when raindrops hit upon tree leaves or falling into a water pool. In addition, for a wide range of applications, such as ink-jet printing, spray cooling, or prevention of soil erosion, droplet impact is of vital importance. The research on this topic already started in 1876 with Worthington, who reported fascinating results observed during droplet impact onto solid and wetted surfaces^1^. Since then, the intriguing and supersizing phenomena of droplet impact dynamics including, fingering, jet and crown formation, receding break-up, and bubble entrapment, to name just a few, continue to attract researchers in the field of physics, mathematics and engineering^2–12^.

Regarding droplet impact onto dry surfaces, besides studies with smooth, rough or textured surfaces, some studies are also devoted to the dynamics of droplets impacting onto solid structures. These studies focus on the droplets bouncing dynamics on curved surfaces^13,14^, dynamics of liquid lamella or sheets after droplet impact onto a small target^15–19^ or close to an edge^20–22^, and droplet capturing^23^ or climbing^24^ on a thin fibre. Studies regarding droplet impact onto a solid sphere have focused on several aspects including droplet spreading and retraction, bouncing dynamics, splashing, and the dynamics of the lamella^25–29^. A number of recent studies have also investigated the droplet impact onto a single stand-alone solid structure embedded upon a surface or onto surfaces with macro-textures^30^. However, most surfaces used here are superhydrophobic surfaces, with the aim of reducing the contact time^31–37^.

In the present study, we investigate droplet impact onto a surface with a stand-alone cubic pillar, through both direct numerical simulations (DNS) and experiments. In contrast to previous studies, here the surface of the pillar and that of the bottom wall satisfy the full wetting condition, which means the contact angle between the liquid fluid and the solid substrate is close to 0°. The simulations, based on a Volume of Fluid method (VOF)^38^, are conducted with our in-house code Free Surface 3D (FS3D)^39^. Results from experiments and computations match very well regarding the droplet morphological changes during the impact. More interestingly, the setup used for the droplet impact leads us to an unexplored interplay between the three phases, the solid structure (cubic pillar), the liquid droplet and the air. We show that, with both experiments and numerical simulations, during the impact process large air bubbles can be entrapped within the fluid flow around the cubic pillar. The numerical simulations further enable to understand the underlying air and bubble entrapment process for our cases, which is intrinsically different from the process occurring on smooth solid surfaces, where the droplet bottom gets deformed by the air that fails to escape, and the contraction of the air film eventually evolves into an air bubble^40–45^. Furthermore, the flow field inside the droplet based on the numerical results have been analysed, and we found a recirculating flow around the entrapped air bubble and high vorticity regions along the pillar surfaces.

Previous studies have discussed the effect of the sharp solid edges and the resulting pinning force on the dynamic wetting of the droplet^46^. In the present study, the pinning of the contact line is not considered in the numerical simulations, because this process occurs only in an extremely short time period for the here investigated Weber and Reynolds numbers and, thus, is negligible for the droplet dynamics investigated within the present study. In addition, for the here investigated cases, both the droplet splashing, and the precursor film^47^ have not been observed during the droplet impact.

## Results and discussion

**Figure 1:**
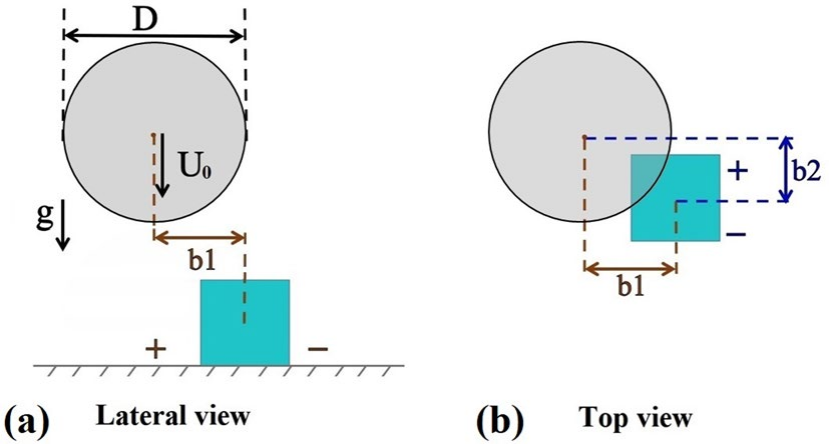
Schematic depiction of a droplet impacting onto a cubic pillar: definition of the droplet impact parameters and the collision parameters $b_1$ and $b_2$ with respect to (**a**) lateral view and (**b**) top view.

This section presents the results from the combined numerical and experimental study: a liquid droplet of about 2 mm in diameter impacts vertically onto a cubic pillar with an edge length of 1 mm × 1 mm × 1 mm. A side view of the impact problem is schematically depicted in Fig. 1a. In the experiment, not only a side view, but also a top view with an inclination of $13.4^{\circ }$ is recorded. From both perspectives, the collision parameters $b_1$ and $b_2$ are extracted, which prescribe the distance between the droplets centre of mass and the centre of the pillar. They are shown schematically in Fig. 1b. Moreover, the bottom view is recorded with the total internal reflection method^48,49^ to analyse the wetted areas around the pillar.

The direct numerical simulation of the droplet impact process is based on a Volume of Fluid method (VOF)^38^. The employed liquid is isopropanol. The liquid properties including the liquid density $\rho$, the dynamic viscosity $\mu$, and the surface tension $\gamma$ are listed in Table 1. In the simulations, we used a static contact angle of 0° between the liquid and the solid phase. In the experiments, both the pillar and the bottom wall are manufactured from acrylic glass (PMMA, Poly(methyl methacrylate)), which exhibits full-wetting properties with regard to the isopropanol droplet. With the help of the sessile drop method, the apparent contact angle of isopropanol on PMMA has been measured. Due to the full wetting properties, no angle was determinable so that 0° has been assumed. This assumption is corroborated by the analytical estimation of the contact angle using the free surface energies of the solid and the liquid phase. The latter are derived from the OWRK-model and the Lewis-acid-base approach^50–53^.

**Table 1:** Physical properties of the test fluid isopropanol at 25 °C.

Liquid	Density, $\rho$ ($\hbox {kg}\cdot\hbox {m}^{-3}$)	Viscosity, $\mu$ ($\hbox {Pa}\cdot\hbox {s}$)	Surface tension, $\gamma$ ($\hbox {N}\cdot\hbox {m}^{-1}$)
Isopropanol	781.5	0.00204	0.02092

In total three cases are considered in this work, including a Near-Centre-Hit (Case 1), a Near-Corner-Hit (Case 2), and an Exact-Centre-Hit (Case 3). The naming of the cases is based on the wetting behaviour of the droplet on the bottom wall. The impact parameters used for all cases, including the impact velocity $U_0$ of the droplet, the droplet diameter *D*, the Weber number $We =\rho U_0^2 D/\gamma$, the Reynolds number $Re = \rho U_0 D / \mu$, and the collision parameters $b_1$ and $b_2$, are listed in Table 2. The first two cases are conducted through both the numerical simulations and the experiments. The Exact-Centre-Hit (Case 3) was investigated by numerical simulation only, since an exact impingement upon the centre of the pillar top can not be realised in the experiment.

**Table 2:** Impact parameters for droplet impact onto a surface with a 1 mm × 1 mm × 1 mm cubic pillar. In all cases full wetting conditions are satisfied. Both numerical simulations and experiments are done for the Case 1 and Case 2. Case 3 is conducted only with the numerical simulation.

	Droplet diameter, *D* (mm)	Impact velocity, $U_0$ ($\hbox {m}\cdot\hbox {s}^{-1}$)	*We*	*Re*	*b*1/*b*2 (mm)
Case 1 *	1.99	1.46	158	1113	− 0.15/− 0.07
Case 2 *	1.99	1.46	158	1113	0.35/− 0.25
Case 3	2.00	1.46	159	1119	0.00/0.00

*Experimental; uncertainty ($$2\sigma$$): $$D\ \pm 3.8\,\%$$, $$U_0\ \pm 1.3\,\%$$.

In the following, we discuss the morphological changes during the droplet impact process. We demonstrate an excellent predictability of our numerical simulations for the macroscopic morphological changes, by comparing the results from both methods for Case 1 and Case 2. Thus, we show through the use of direct numerical simulations to deeper explore physical processes for the conducted cases as it is possible in most experimental methods. Afterwards, we discuss, based on the evolution of the interfaces, the process of the air entrapment and the evolution of the entrapped air into bubbles. Finally, to gain more insight into the droplet dynamics and the flow physics, we discuss for Case 3 (the Exact-Centre-Hit) the microscopic flow features inside the droplet, based on the computational results.

### Comparison of macroscopic morphology

**Figure 2:**
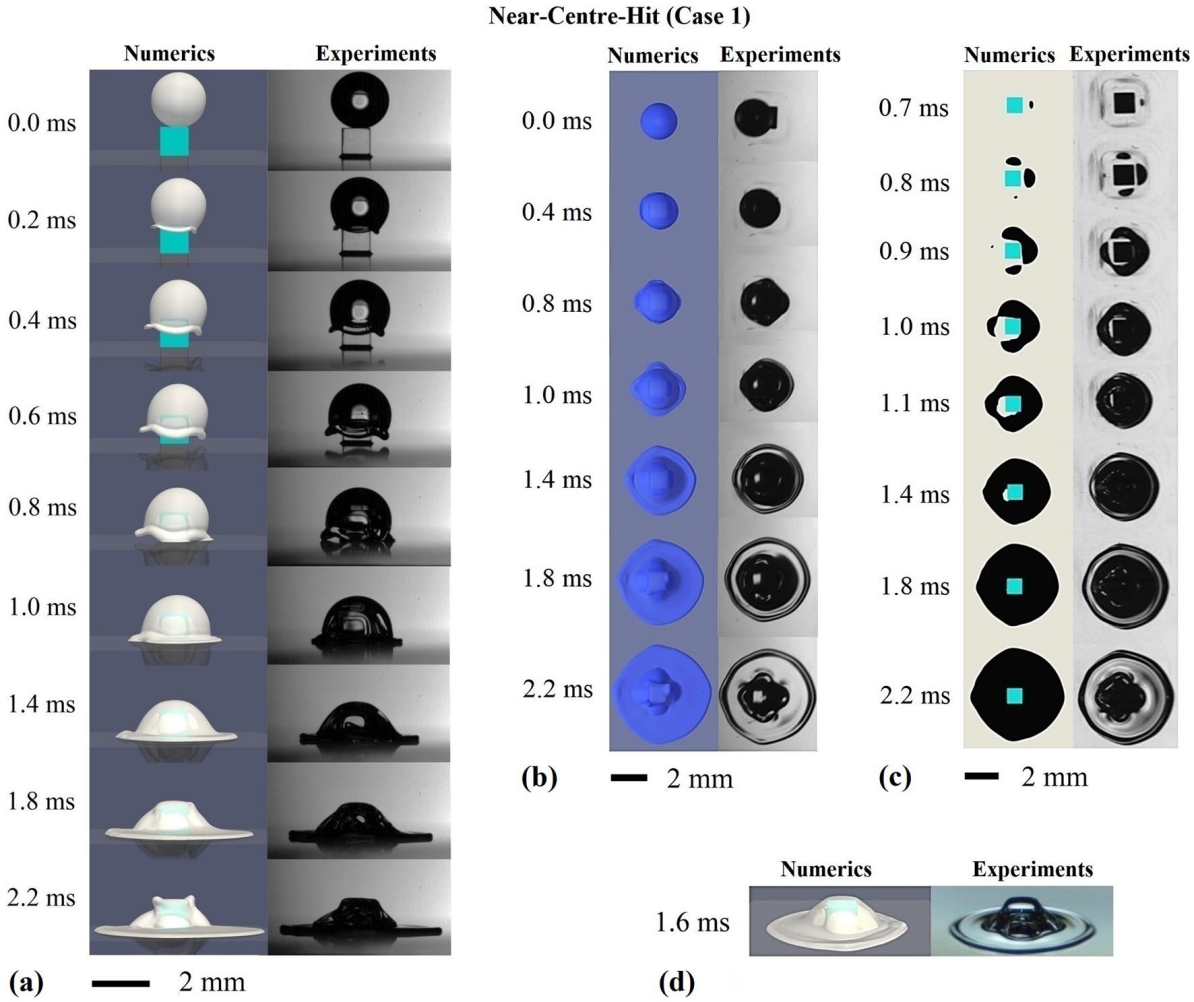
Photographs (right) and direct numerical simulation results (left) regarding the impingement of an isopropanol droplet of about 2 mm in diameter onto a 1 mm × 1 mm × 1 mm cubic pillar embedded on a surface. Surface of the pillar and the bottom wall satisfy the full-wetting condition. $We = 158$, $Re = 1113$. The collision parameters *b*1 and *b*2 are − 0.15 and − 0.07, respectively. (**a**) side view; (**b**) top view; (**c**) bottom view; (**d**) transparent side view. The top view is recorded with an inclination of $13.4^{\circ }$.

**Figure 3:**
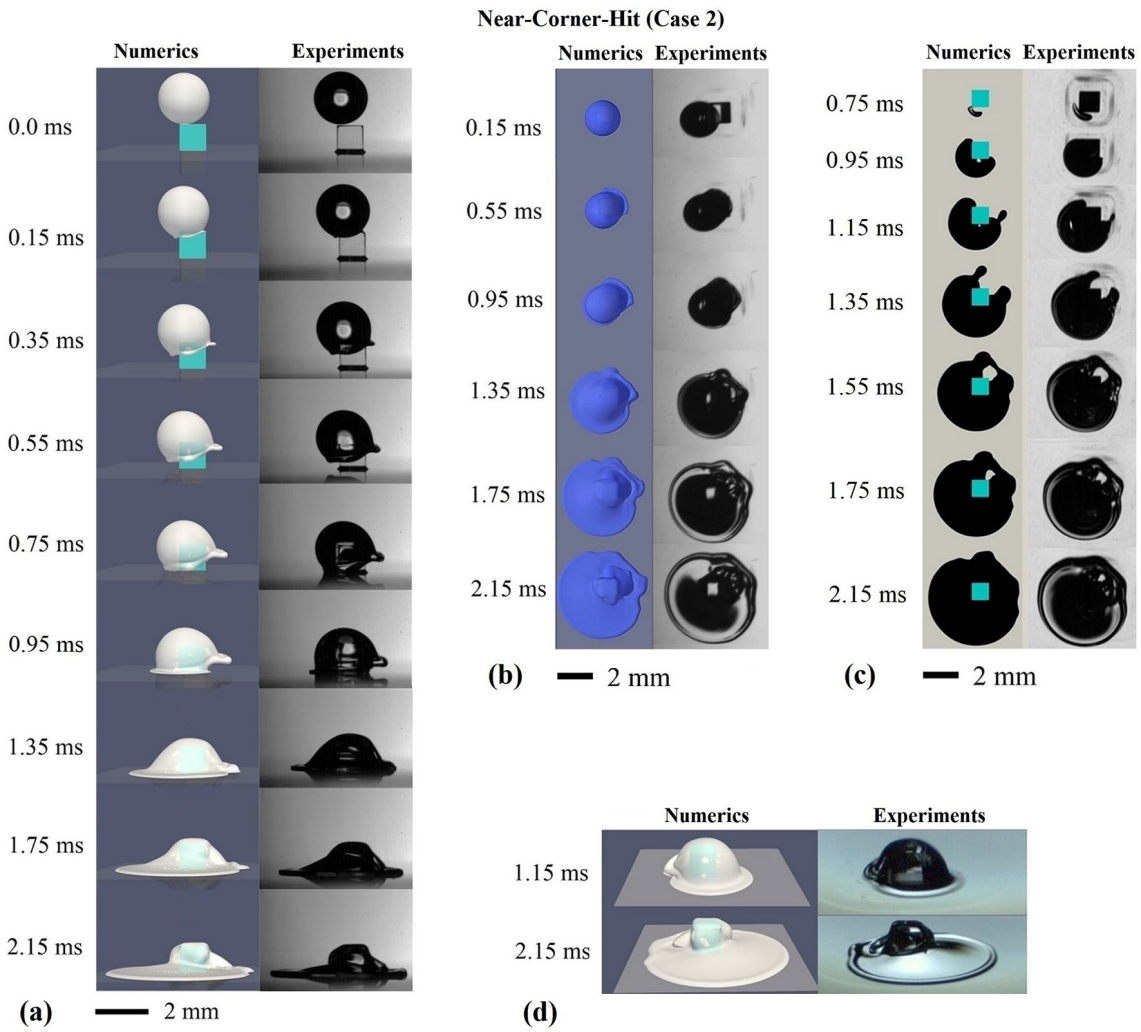
Photographs (right) and direct numerical simulation results (left) regarding the impingement of an isopropanol droplet of about 2 mm in diameter onto a 1 mm × 1 mm × 1 mm cubic pillar embedded on a surface. Surface of the pillar and the bottom wall satisfy the full-wetting condition. $We = 158$, $Re = 1113$. The collision parameters *b*1 and *b*2 are 0.35 and -0.25, respectively. (**a**) side view; (**b**) top view; (**c**) bottom view; (**d**) transparent side view. The top view is recorded with an inclination of $13.4^{\circ }$.

Figures 2 and 3 show sequences of images for the droplet impact process of a Near-Centre-Hit (Case 1) and a Near-Corner-Hit (Case 2), respectively. The results in the left columns are obtained from the numerical simulations, and the right columns from the experiments. In the Near-Centre-Hit, the droplet is impacting slightly right to the pillar centre, and in the Near-Corner-Hit close to the left side of the pillar. In both cases the droplets centre of mass at $t=0.0\,\mathrm{ms}$ is located in the front side of the pillar centre, corresponding to the definition of *b*1 and *b*2 in Fig. 1.

The images from the side view in Fig. 2a reveal, in the case of the Near-Centre-Hit, the details on the droplet spreading behaviour as well as the dynamics of the droplet rim during impact. Shortly after the droplet impacts onto the pillar, the spreading rim upon the pillar top is expelled away from the edges of the pillar ($t = 0.2\,\mathrm{ms}$). Due to the geometry of the pillar, the droplet rim at the edges of the pillar expands earlier than the rim at the corners of the pillar. Over time the droplet spreads beyond the pillar and takes the form of a round cap surrounded by the undulated droplet rim. The rim thickens due the influence of the surface tension force ($t=0.2\,\mathrm{ms}{-}0.6\,\mathrm{ms}$). The slight shift of the cap-like droplet towards the right side is induced by the distance of the collision point from the centre of the pillar top (see Table 2). Thereby, the rim at the right edge of the pillar, which is closest to the collision point, first wets the bottom wall and vice versa ($t = 0.8\,\mathrm{ms}$). Afterwards, the droplet continues to spread on the bottom wall, and transforms gradually from the shape of a round cap into a straw hat ($t = 1.0\,\mathrm{ms}{-}2.2\,\mathrm{ms}$). Compared to the experiments, the computational results in the side view display here a very similar droplet impact process with respect to the overall droplet deformation including the redirection of the rim through the pillar, the thickening of the rim ($t = 0.2\,\mathrm{ms}{-}0.8\,\mathrm{ms}$), and later on the spreading of the droplet.

The bottom perspective of the experiments in Fig. 2c (right column) displays that, in the experiment the wetting of the bottom wall around the pillar starts from one side of the pillar ($t = 0.7\,\mathrm{ms}$), followed by an almost symmetrical wetting along the two neighbouring pillar sides ($t = 0.8\,\mathrm{ms}$). Subsequently, the wetting spreads to the remaining pillar side ($t=0.9\,\mathrm{ms}$), until finally the wetted area surrounds the pillar completely. The wetting occurs first at the edges of the pillar and then the liquid closes in on the corners, resulting in a square shape of the wetted area at ($t=0.9\,\mathrm{ms}$), rotated by about $45^{\circ }$ with respect to the pillar edges. Later on ($t>1.1\,\mathrm{ms}$), the droplet spreads more evenly away from the corners of the pillar, resulting in a shape close to a square with rounded edges at $t=2.2\,\mathrm{ms}$. The computational results (Fig. 2c, left column) display a sequence of the wetting process, with the shape and size of the wetted area similar to the experiments, especially in a late time stage (after 1.1 ms). Some deviations in the propagation of the wetting at earlier times are dominated mainly by the uncertainties in the measurement of *b*1 and *b*2 (see method section). The top views (Fig. 2b) from both methods also display similar changes in the shape of the droplet during its impact. The top view images from both methods display in the latest time step ($t = 2.2\,\mathrm{ms}$) a similar degree of curvature and a very close distribution of the curved area on the droplet surface. These curved areas indicate, that large volumes of air or air bubbles are trapped underneath the droplet, which will be discussed in more details later. The transparent lateral view in Fig. 2d clearly shows that there is air trapped underneath the droplet. Results from both methods are qualitatively very similar in terms of both the position and the volume of the air around the pillar.

In the case of the Near-Centre-Hit (Fig. 2), the fluid in the rim of the impacting droplet expands away from the pillar top before hitting upon the bottom wall. This is due to the fact that the fluid momentum in the droplet rim transfers from the vertical direction to the horizontal direction. In contrast, in case of the Near-Corner-Hit (Fig. 3), the fluid at the pillar edges, close to the collision point, is dominated by the momentum of the droplet in the vertical direction. This explains why in Fig. 3a the fluid in the droplet rim around the corner and the edges closer to the collision point moves straight down towards the bottom wall, without being expelled away from the pillar. Furthermore, the rim at the edges further away from the collision point expands even stronger away from the pillar ($t=0.75\,\mathrm{ms}, 0.95\,\mathrm{ms}$ in Fig. 3a), compared to the Near-Centre-Hit ($t=0.6\,\mathrm{ms}, 0.8\,\mathrm{ms}$ in Fig. 2a). Due to the expansion of the rim at one side, the droplet deforms into a shape of a peaked cap ($t = 0.35\,\mathrm{ms} - 0.75\,\mathrm{ms}$). The spreading on the bottom wall starts from the left side and continues on both sides of the pillar after the expanded rim reaches the bottom wall. At a late-time stage, e.g. $t = 2.15\,\mathrm{ms}$, the spreading is observed to be more profound on the left side of the pillar (close to the collision point). Results from both experiments and numerics again display similar dynamics of the rim with regard to both the changes of the rim direction and the thickening of the rim over time. Moreover, the time dependent droplet shape and the spreading of the droplet on the bottom wall, which is governed by both the inertial and the viscous forces, match very well in both methods.

The bottom views from both methods in Fig. 3c also display a very similar wetting process on the bottom wall. The wetting of the bottom wall starts at the pillar corner that is closest to the collision point ($t = 0.75\,\mathrm{ms}$), followed by the wetting around the two neighbouring pillar sides ($t = 0.95\,\mathrm{ms}$ to $t = 1.35\,\mathrm{ms}$). The closing of the wetted area around the pillar starts after the strongly repelled rim hits the bottom wall at the opposite side of the impact point (around $t = 1.55\,\mathrm{ms}$). Eventually the whole bottom area around the pillar gets wetted. The top view in Fig. 3b displays more details on the shape changes of the droplet over time, which are reproduced very well by the numerical simulation. Moreover, also in the case of the Near-Corner-Hit, the latest time step ($t=2.15\,\mathrm{ms}$) from both methods displays a similar distribution of the curved area on the droplet surface, indicating that the position and the size of the large bubble trapped underneath the droplet are comparable in both methods. Despite some deviations in the propagation of the wetted area around the pillar (e.g. $t = 1.15\,\mathrm{ms}$ to $t = 1.55\,\mathrm{ms}$), mainly due to the uncertainties in the collision parameters (see method section), the main features of the droplet morphological changes as well as the entrapped air are very well captured by the numerical simulations.

In Case 1 and Case 2, we discussed the difference in the morphological changes of the impacting droplet, especially with respect to the droplet spreading, wetting progress, the deflection of the rim from the pillar as well as the shape change of the droplet. For both cases, we have compared the computational results with the experimental results in three different perspectives. The dynamics of the impacting droplet obtained from the experiments and the direct numerical simulations based on a Volume of Fluid method (VOF)^38^ matches very well.

### Air entrapment and successive bubble formation process

**Figure 4:**
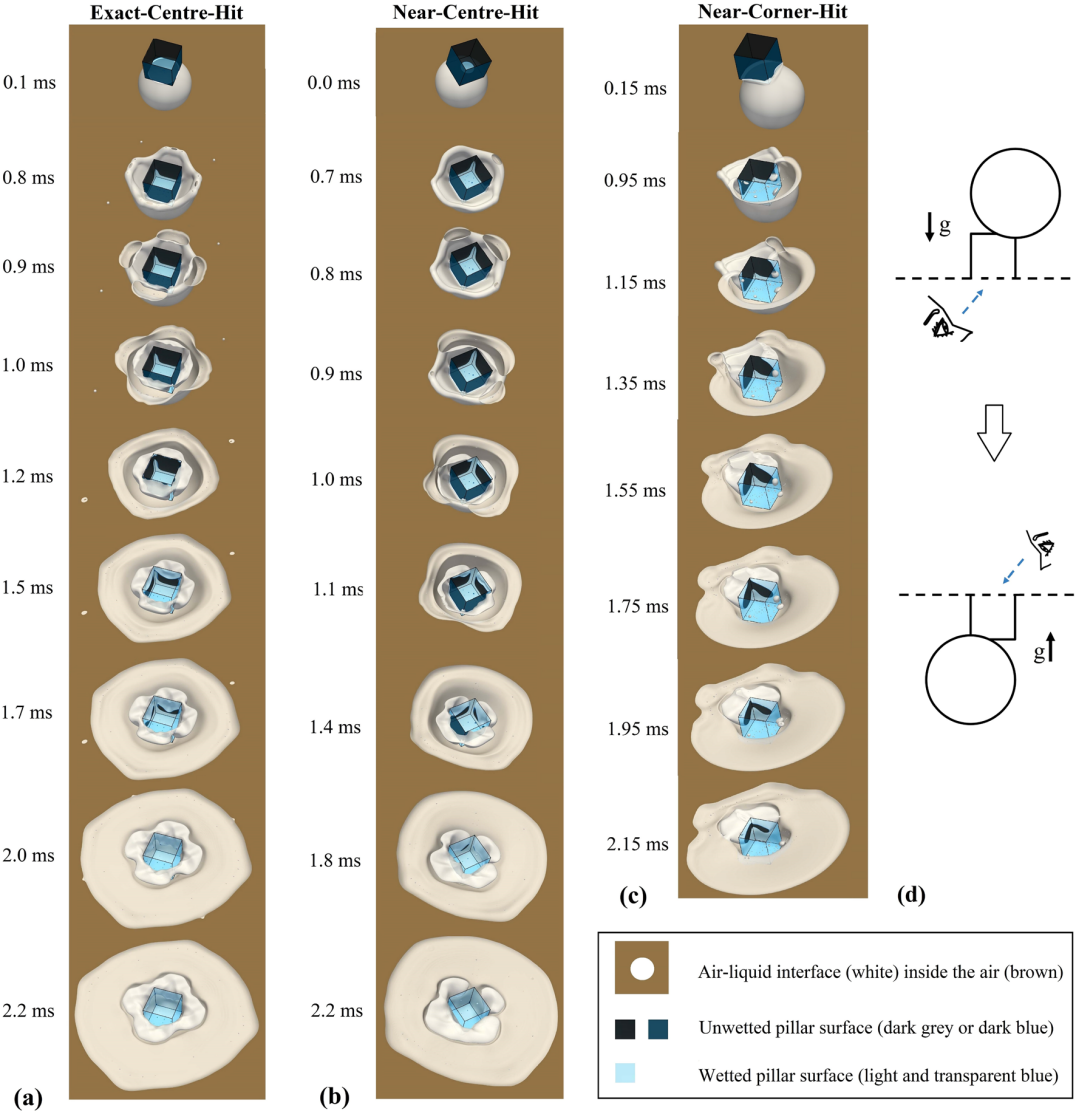
Bottom views showing the process of air entrapment and consequent transfer into air bubbles. Results are extracted from the direct numerical simulations. White colour denotes the interface between the air and the fluid; transparent and light blue indicate the wetted area on the pillar surface; dark grey and dark/prussian blue indicate the unwetted area on the pillar surface. (**a**): Exact-Centre-Hit (Case 3); (**b**): Near-Centre-Hit (Case 1); (**c**): Near-Corner-Hit (Case 2). (**d**): Schematic of the observation method for the results in (**a**), (**b**), and (**c**).

Before, we have observed that the droplet rim can expand away from the pillar, due to the transformation of the momentum from the vertical to the horizontal direction. The rim, at the edges further away from the collision point, gets deflected strongly away from the pillar. At the corner and pillar edges close to the collision point, the flow in the rim is dominated by the droplets vertical momentum, such that the fluid travels straight down towards the bottom wall without being deflected outwards. If and how strongly the rim is deflected away from the pillar are important for the wetting of the corners and side faces of the pillar, the volume and the location of the air trapped around the pillar, as well as for the transfer of the entrapped air into bubbles. The detailed process is presented in Fig. 4, through the bottom perspective using the results from the direct numerical simulations. Fig. 4d depicts schematically the observation method. The transparent bottom wall and pillar faces as well as an oblique view angle from the bottom (for each case) enable clear visualisation of the three-dimensional air entrapment process. In Fig. 4a,b,c, the white colour denotes the interface between the air and the fluid, transparent and light blue indicate the wetted area on the pillar surface, and dark grey and dark/prussian blue indicate the unwetted area on the pillar surface.

In the Exact-Centre-Hit case (Case 3), the droplet spreading behaviour, including the dynamics of the droplet rim at early times, is similar to the Near-Centre-Hit case (Case 1). In both cases, the droplet rim expands after the impingement away from the four edges of the pillar top. However, in Case 3 where the collision point lies exactly at the centre of the pillar, the expansion of the droplet rim and later on the wetting and spreading process on the bottom wall is fourfold symmetric with respect to the pillar. Figure 4a ($t = 0.8\,\mathrm{ms}$) displays the Exact-Centre-Hit case (Case 3) from a bottom perspective. The spreading droplet on the pillar can be seen together with the propelled droplet rim away from the pillar edges. In the meantime, more than half of the area of the pillars vertical edges has been wetted by the droplet liquid while the four side faces of the pillar are barely wetted due to the expanding rim. The trapping of the air around the cubic pillar and underneath the impacting droplet starts as the propelled rim reaches the bottom wall. The fluid in the rim at the bottom wall propagates in both directions, away from the pillar and towards the bottom edges of the pillar ($t = 0.8\,\mathrm{ms}$ to 1.2 ms). At $t=1.2\,\mathrm{ms}$, the fluid of the inner rim reaches the bottom sides of the pillar and starts to wet the side faces of the pillar ($t = 1.2\,\mathrm{ms}$ to $t=1.7\,\mathrm{ms}$), which is indicated by the colour change from dark grey to transparent blue. This also illustrates how the entrapped air is evolving into the air bubbles, that detach from the side walls of the pillar over time. Larger volumes of air are trapped at the pillar side faces than at the pillar corners. This is due to the fact that the deflection of the rim away from the pillar is stronger at the pillar side faces than at the corners. Interestingly, one also observes that as the fluid wets the pillar sides, the wetting of the fluid as well as the detachment of the air happen faster at the centre of the side faces ($t=1.5\,\mathrm{ms}$ and $t = 1.7\,\mathrm{ms}$). The air bubble detachment is important, since this process involves the creation of surfaces (energy) and can therefore influence, the dynamics of the bubble as well as the surrounding flow field. We will continue to investigate the flow inside the droplet for Case 3 in the next section, including the velocity and the vorticity field.

Figure 4b illustrates the air entrapment process for the case of the Near-Centre-Hit (Case 1). As shown in Fig. 4b and Fig. 4a ($t = 2.2\,\mathrm{ms}$), both the Near-Centre-Hit and the Exact-Centre-Hit show that the entrapped air eventually surrounds the four side faces of the pillar. However, in the Near-Centre-Hit one separate small bubble is observed at the pillar side closest to the collision point. At the side furthest away from the collision point, the highest volume of air is entrapped. This is due to the fact, that the rim at the edges further away from the collision point gets deflected strongly away from the pillar, leading to more space for the air between the pillar and the liquid-air interface before the rim reaches the bottom wall. Compared to the Exact-Centre-Hit (Case 3), the air entrapment process of the Near-Centre-Hit (Case 1) is rather similar and is summarised as follows: In Case 1, after the droplet rim reaches the bottom wall, the inner rim propagates towards the pillar bottom ($t = 0.7\,\mathrm{ms}$ to $1.1\,\mathrm{ms}$). In the meantime, the fluid from above already wets most area of the vertical edges, but barely wets the side faces of the pillar. After reaching the bottom of the pillar edges, the inner rim of the droplet starts to wet the side faces of the pillar ($t = 1.1\,\mathrm{ms}$ to $1.8\,\mathrm{ms}$), illustrating the detachment of the air from the bottom wall and the pillar side faces. In contrast to the Exact-Centre-Hit (Case 3), in the Near-Centre-Hit (Case 1) the fluid from above wets the two corners of the pillar near to the collision point faster. As shown in Fig. 4b, by $t = 1.0\,\mathrm{ms}$ the vertical pillar edge closest to the collision point is fully wetted, followed by the full wetting of the neighbouring vertical edge ($t = 1.4\,\mathrm{ms}$). This eventually leads to the formation of a separate air bubble.

Unlike the Exact-Centre-Hit (Case 3), where the entrapped air surrounds four side faces of the pillar, in the Near-Corner-Hit (Case 2) a large volume of air is trapped at the corner furthest away from the collision point (Fig. 3b). As in Fig. 3a, shortly after the droplet impingement, the fluid propagates quickly towards the vertical edge of the pillar closest to the collision point and its two neighbouring side faces ($t = 0.15\,\mathrm{ms} - 0.95\,\mathrm{ms}$). By $t = 0.95\,\mathrm{ms}$, these two side faces and the vertical edge in between are almost fully wetted by the fluid (Fig. 4c). As shown in Fig. 4c, after $t = 0.95\,\mathrm{ms}$ the droplet rim at the bottom edges of the pillar propagates further along the unwetted sides of the pillar. Whereas the expanding rim in the air (the outside rim) starts to wet the bottom wall at around $t = 1.15\,\mathrm{ms}$ from one side, followed by the wetting from the other side of the rim at around $t = 1.35\,\mathrm{ms}$. By $t = 1.55\,\mathrm{ms}$, the fluid from the expanding rim has all reached the bottom wall and a large volume of air is trapped within the droplet. The displacement of the fluid from the inner and the outside rims ($t = 0.95\,\mathrm{ms}$ to $1.55\,\mathrm{ms}$) shows that, the trapping of such a large volume of air becomes possible, when the outside rim reaches and wets the bottom wall before the space underneath is vastly invaded by the fluid from the inner rims. From $t = 1.55\,\mathrm{ms}$ to $2.15\,\mathrm{ms}$, the wetting at the bottom wall and that on the side faces of the pillar continues, illustrating how the entrapped air evolves into the air bubble and starts to detach from the bottom wall and the pillar surfaces.

### Microscopic flow features

**Figure 5:**
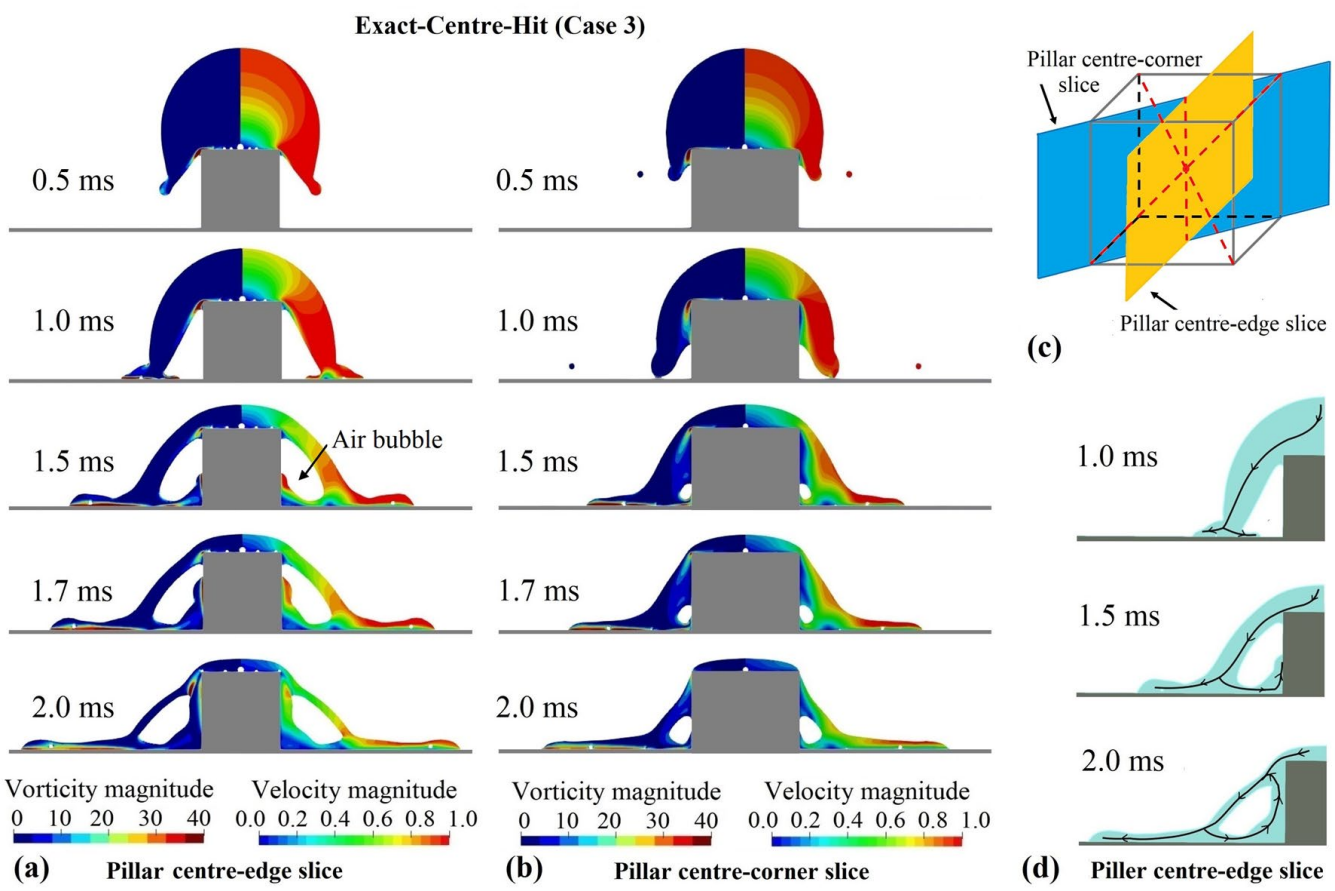
Flow field of a $D = 2\,\mathrm{mm}$ isopropanol droplet, impacting onto a 1 mm × 1 mm × 1 mm cubic pillar embedded on a flat surface. The collision point lies at the centre of the pillar top. The contact angle is $0^{\circ }$, satisfying the full-wetting condition. $We = 159$ and $Re = 1119$. (**a**) Dimensionless vorticity field $2D\left| \omega \right| /\ U_0$ (left) and dimensionless velocity field $\left| V \right| /\ U_0$ (right) for a slice across the pillar centre and the centre of the pillar edges. (**b**) Dimensionless vorticity field $2D\left| \omega \right| /\ U_0$ (left) and dimensionless velocity field $\left| V \right| /\ U_0$ (right) for a slice across the pillar centre and the corners of the pillar. (**c**) Position of the two slices in (**a**) and (**b**) through the cubic pillar. (**d**) Schematic streamline direction for the same slice as shown in (**a**).

The flow field inside the impacting droplet of the Exact-Centre-Hit case (Case 3) is illustrated in Fig. 5. Figures 5a and 5b present the non-dimensionalised velocity magnitude $\left| V \right| /\ U_0$ (right column) and the non-dimensionalised vorticity field $2D\left| \omega \right| /\ U_0$ (left column) of the droplet in two intersecting slices, with one slice across the centre of the pillar sides and another across the corners of the pillar. Here, *V* denotes the velocity field and $\omega = \nabla \times \ V$. The position of the two slices are shown schematically in Fig. 5c. Corresponding to the results in Fig. 4a, Fig. 5a,b display that after the droplet rim reaches the bottom wall, part of the fluid spreads towards the cubic pillar. In the current case, the droplet rim has just enough energy to climb up the side faces of the pillar and form a closure of the fluid around the entrapped air bubble. The white area inside the droplet indicates that the air bubble is still fully connected around the pillar corner. The volume of air at the corner is much smaller than at the central side of the pillar.

The velocity field in Fig. 5a, b displays clearly the deceleration of the flow at the stagnation points, with the first stagnation point at the centre of the pillar top. The second stagnation point is at the first contact point of the droplet rim with the bottom surface. Near both the bottom wall and the pillar surface, the flow velocity decreases to zero due to the no-slip condition. Correspondingly, the vorticity magnitude increases dramatically. A sequence of the streamline direction for the same slice as in Fig. 5a is shown schematically in Fig. 5d. The streamline direction illustrates the completion of one internal circulation of the fluid flow around the entrapped air.

**Figure 6:**
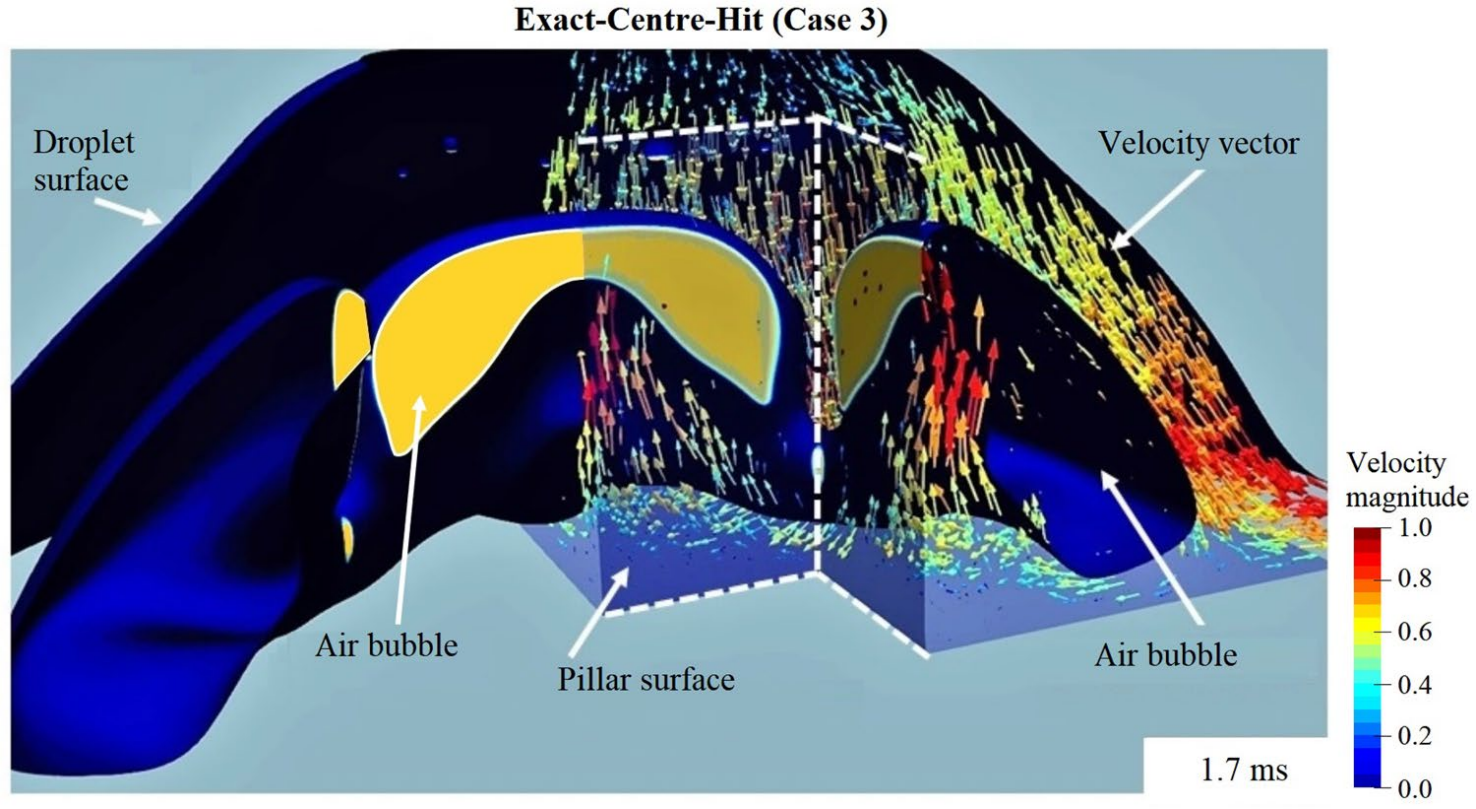
3D velocity vector field of the droplet at one corner of the pillar. The colour of the velocity vector depicts the dimensionless velocity magnitude $\left| V \right| /\ U_0$. Results from a $D=2\,\mathrm{mm}$ isopropanol droplet, impacting onto a 1 mm × 1 mm × 1 mm cubic pillar embedded on a flat surface. The collision point lies at the centre of the pillar top. The contact angle is $0^{\circ }$, satisfying the full-wetting condition. $We = 159$ and $Re = 1119$. The edges of a quarter of the cubic pillar are outlined with white dashed lines for visualisation purpose. Both the droplet and the bubble surface (interface between the air and the liquid) are denoted by navy blue; transparent blue of the pillar indicates the wetted area on the pillar surface; the yellow area is part of the air bubble and indicates the unwetted area on the pillar surface. The velocity vector field as well as the surface of the droplet, the air bubble and the pillar were generated with free software Paraview 5.9.0 (www.paraview.org).

Figure 6 presents a snapshot of the 3D velocity vector field inside the droplet at $t = 1.7\,\mathrm{ms}$. The velocity vector field reveals that at the pillar corner the reversed flow towards the pillar is driven away by the sharp vertical edge of the pillar, leading to a stronger flux at the vertical symmetry axis of the pillar side faces. This explains the phenomena observed in Fig. 4a ($t = 1.5\,\mathrm{ms}$ and 1.7 ms) that at the centre of the side faces, the wetting of the fluid as well as the detachment of the air bubble take place faster.

## Conclusion

The impact of an isopropanol droplet onto a cubic pillar embedded on a flat surface has been studied. Very good agreement concerning the droplet morphological changes has been found between the experimental and the numerical results based on a Volume of Fluid method^38^. A large volume of air is observed to be trapped around the pillar side faces. The collision point on the pillar strongly influences the position and the size of the entrapped air. Based on the computational results, the three-dimensional process for the trapping of air underneath the droplet and the evolving of air into bubbles are unravelled. This shows nicely how direct numerical simulations of multiphase flow can be used to further support our understanding of complex physical phenomena, like the air entrapment during droplet impingement.

## Methods

### Direct numerical simulations based on a volume of fluid (VOF) method

The droplet impact process is computed with our in-house code Free Surface 3D (FS3D), a program for direct numerical simulations of incompressible multiphase flow. The program, which was initially developed by Rieber^54^ in the late 1990s for splashing simulations has been successfully applied for studies of a wide range of problems in multiphase flow, including binary droplet collisions^55^, droplet impact onto macro-structures^37^, droplet in turbulent air flow^56^, non-Newtonian jet breakup^57^, and evaporating droplets^58,59^. Interested readers may refer to the overview on FS3D by Eisenschmidt et al.^39^ for more details. In the following, we give a brief summary of the methods used in the simulations.

We assume, in the present study, the impact of an isopropanol droplet onto a cubic pillar as isothermal. The flow field is thereby governed by the incompressible Navier-Stokes-equations (NS) for the conservation of mass and momentum. The tracking of the interface between different phases is based on the Volume of Fluid method (VOF)^38^. Hereby, the volume fraction of the liquid phase *f* is introduced as

1 $$\begin{aligned} f(\mathbf {x},t)=\left\{ \begin{array}{ll} 0 &{}\quad {\text {outside the liquid region,}}\\ 0<f<1 &{} \quad {\text {at the interface,}}\\ 1 &{} \quad {\text {inside the liquid region.}} \end{array}\right. \end{aligned}$$

The transport equation for *f* reads

2 $$\begin{aligned} f_t+\nabla \cdot [f\mathbf {u}]=0, \end{aligned}$$

where $f_t$ denotes the partial derivative of *f* with respect to time and **u** the velocity vector. For the spatial discretization, the finite volume method on the basis of a MAC staggered grid^60^ is used. To minimise the numerical diffusion, the interfaces are reconstructed by Piecewise Linear Interface Calculation (PLIC)^61^. A second order accurate Strang-splitting scheme is used for both the liquid volume advection and the momentum advection^62^. The surface tension force in the momentum equation is modelled as a volume force via the Continuous Surface Stress (CSS) model^63^. For the time integration, an explicit Euler scheme is used. More details on the numerical methods regarding e.g. the treatment of the contact angle are to be found in a previous study on the drop impact on a superhydrophobic surface with a wire^37,64^.

Our numerical setup consists of a spherical droplet of about 2 mm in diameter falling onto a stand-alone 1 mm × 1 mm × 1 mm cubic pillar placed on a flat surface. We adopt a contact angle of 0°, corresponding to the full wetting condition in the experiments. In this study, the droplet impact is simulated in a three-dimensional rectangular domain using an equidistant Cartesian mesh. On the surface of the bottom wall and the cubic pillar, no slip boundary conditions are applied. On the rest of the boundaries of the computational domain, continuous boundary conditions (Neumann) are used. In all simulation cases, the edges of the cubic pillar are placed exactly at the cell edges. In Case 1 and Case 3, where the collision points lie near to or exactly at the centre of the pillar top, a grid resolution of about 250 grid cells per droplet diameter *D* (grid cell sizes of about $8\,\upmu \hbox {m}$) is used, which guarantees both the convergence of droplet morphology as well as the flow field of the internal flow. In Case 2, where the collision point lies further away from the pillar centre, 304 grid cells per droplet diameter (grid cell sizes of about $6.5\,\upmu \hbox {m}$) is used. The dimension of the domain is about 2*D* × 4*D* × 4*D* in Case 1 and Case 3, and about 1.6*D* × 3.2*D* × 3.2*D* in Case 2, resulting in a total of about 0.5 billion grid cells for each case. The used numbers of grid cells have been investigated before in a grid sensitivity study and have been found to be suitable for the predictions shown here.

### Experimental method

The impact of an isopropanol droplet onto a single, free-standing pillar (1 mm × 1 mm × 1 mm) is investigated experimentally with a multi-perspective (top, bottom, lateral and spatial view) imaging approach.

The associated experimental test facility consists of four main components, which are the droplet generation unit, the surface specimen, the image acquisition unit, and the triggering and synchronisation unit. A detailed description of the facility and the post-processing procedures can be found in^49^.

#### Droplet generation unit

The droplets are generated with a blunt tilted needle, which is fed by a medical syringe pump. All tubes, syringes and connectors, are either made of Teflon or glass or are medical equipment, in order to avoid any chemical interactions between the fluids and the tubing system. A constant droplet chain is established of which one random droplet is selected to pass the droplet barrier. The droplets impact velocity can be selected by adjusting the falling height of the droplet.

#### Surface specimen

The surface specimen including the free-standing pillar are manufactured of acrylic glass using very precise milling-process. In general, the pillar dimensions comply very well with the predefined edge lengths of 1 mm. However, due to the manufacturing process, the pillar base has a radius of about 0.1 mm. Nevertheless, all experiments confirmed, that there is no significant influence on the droplet impact morphology. Furthermore, the combination of acrylic glass and isopropanol shows a full wetting behaviour.

#### Image acquisition unit

The lateral and top view perspective of the droplet impact are captured with a classical diffuse back-light imaging technique. The top view perspective is recorded at an angle of $13.4^{\circ }$ to avoid an obstruction of the image by the exit of the droplet generation unit. The bottom perspective is recorded in a total internal reflection mode^48,65^, which allows to distinguish dry and wetted areas on the target surface. The fourth perspective observes the impact process from an inclined position in order to obtain a spatial view, which gives a three-dimensional impression of the scenery. This perspective helps to distinguish and compare small features with the numerical simulations, e.g. air entrapment, finger formation or secondary droplets.

#### Triggering and synchronisation unit

The image acquisition and, therefore, all cameras are trigger with a LASER light barrier. Before impact, the selected droplet passes this barrier and a TTL-signal triggering the cameras is generated. The three main perspectives, top, bottom and lateral view, are acquired by two fully synchronised Photron SA-X2 cameras. The top and lateral view are recorded with one camera, while the second camera records the bottom view. The applied resolution for both cameras is $1024 \times 672\,\hbox {px}^{2}$ at a shutter speed of 1/88888 s and a frame rate of 20000 fps. The utilised optical resolutions are $17\,\upmu \hbox {m}/\hbox {px}$, $18\,\upmu \hbox {m}/\hbox {px}$, and $28\,\upmu \hbox {m}/\hbox {px}$ for the bottom, lateral, and top view, respectively. The spatial view is recorded with a Chronos 1.4 colour high-speed camera having a resolution of $1280 \times 1024\,\hbox {px}^{2}$ at a shutter time of $115.5\,\upmu \hbox {s}$ and a frame rate of 1000 fps. The corresponding optical resolution of this perspective is $11\,\upmu \hbox {m}/\hbox {px}$. The Chronos 1.4 camera is triggered with the other two cameras, however, it is running independently from the internal clocks of the Photron cameras.

Before the images of the top, bottom, and lateral perspectives are used for any evaluation, they are individually corrected to avoid any perspective distortion using previously generated calibration measurements and bicubic interpolations.

With the help of an image processing routine^49^, the droplet impact diameter and velocity are determined with an accuracy of 3.8 % ($2\sigma$) and 1.3 % ($2\sigma$), respectively. Thereof, the uncertainty of the Reynolds and Weber numbers are 4.0 % and 4.6 %, respectively. The collision parameters $b_1$ and $b_2$ are evaluated manually from the last image before the droplet impacts onto the pillar. Hereby, the centroid position of the droplet with respect to the position of the pillar have been used. The parameter $b_1$ is extracted from the lateral perspective, due to the higher optical resolution, while $b_2$ can only be determined from the top perspective. The uncertainty for the parameters are 2 % for $b_1$ and 12 % for $b_2$ with respect to the pillar width of 1 mm.

## Data Availability

The datasets generated during the current study are available from the corresponding author on reasonable request.
